# The Influence of *Streptococcus mutans* Biofilm Formation in a Polymicrobial Environment ( *Streptococcus gordonii* & *Porphyromonas gingivalis* )

**DOI:** 10.1055/s-0044-1782215

**Published:** 2024-05-14

**Authors:** Indah Listiana Kriswandini, Sidarningsih Sidarningsih, Adelheid Chrissanda Hermanto, Pinta Rahayuning Tyas, Mohammed Ahmed Aljunaid

**Affiliations:** 1 Department of Oral Biology, Faculty of Dental Medicine, Universitas Airlangga, Surabaya, Indonesia; 2 Student of Dental Medicine, Faculty of Dental Medicine, Universitas Airlangga, Surabaya, Indonesia; 3 Department of Dental Medicine, Faculty of Medicine, Taiz University, Taiz, Yemen

**Keywords:** *Streptococcus mutans*, quorum sensing, infectious disease, biofilm, caries

## Abstract

**Objectives** Biofilms play a vital role in the occurrence or worsening of an infectious disease. *Streptococcus mutans* is a bacterium with the ability to form biofilms that plays a key role in the development of infectious diseases such as dental caries. The formation of biofilms in *S. mutans* is mediated by quorum sensing. Inhibiting quorum sensing can be considered as one of the approaches to prevent caries. This study aims to investigate the ability of *Streptococcus gordonii* and *Porphyromonas gingivalis* bacteria to inhibit the formation of *S. mutans* biofilm.

**Materials and Methods** This research was conducted to analyze bacterial biofilm formation and metabolism. The bacteria used are *S. mutans* (serotype C), *S. gordonii* (ATCC 5165), and *P. gingivalis* (ATCC 33277). Biofilm formation was analyzed using the crystal violet assay. Bacterial metabolism was analyzed using the methylthiazol tetrazolium (MTT) assay.

**Results** The results of the crystal violet assay indicate a decrease in biofilm formation in *S. mutans* when in the presence of *S. gordonii* and *S. mutans* in the presence of *P. gingivalis* . The results of the MTT assay show no significant change in the bacterial metabolism of *S. mutans* in the presence of *S. gordonii* and *S. mutans* in the presence of *P. gingivalis* . However, *S. mutans* with the presence of *S. gordonii* and *P. gingivalis* show an increase in biofilm formation and bacterial metabolism.

**Conclusion** *S. gordonii* and *P. gingivalis* are each capable of inhibiting the formation of *S. mutans* biofilm in a polymicrobial environment.

## Introduction

Biofilm has now been proven to be associated with and play a vital role in the occurrence or worsening of an infectious disease. According to research conducted by The National Institutes of Health, among all microbial infections and chronic infections, 65 and 80% of them, respectively, are related to biofilm formation. 1 Within the oral cavity, there are more than 700 species of bacteria. 2 These bacteria communicate with each other through quorum sensing. Quorum sensing is a mechanism of microbial communication in response to environmental changes such as nutrient availability and bacterial density. Bacteria will synthesize signaling molecules called autoinducers, and when these autoinducers reach a threshold or “quorum” concentration, bacterial biofilms will recognize the presence of these autoinducer components and undergo changes in gene expression and behavior within the biofilm. 3 4

*Streptococcus mutans* is one of the flora normal bacteria of oral cavity and serves as the main agent in the formation of dental caries. *S. mutans* has the ability to form biofilms on the tooth surface. The ability of *S. mutans* to form biofilms is crucial for its survival and the development of caries. These formed biofilms can persist in the oral cavity, leading to the progression of dental caries if not properly managed. 5 Several factors contributing to *S. mutans'* ability to form and maintain biofilms in the oral cavity include its ability to survive in acidic environments, interactions with other microorganisms, and the production of polysaccharides that encase the biofilm. 6 7

Biofilm formation of *S. mutans* is mediated through quorum sensing, facilitated by competence stimulating peptide (CSP) via the two component pathway (ComDE). CSP is responsible for regulating the transcription of specific target genes in biofilm formation, namely, glucosyltransferase B/C/D ( *gtf B/C/D* ), fructosyltransferase ( *ftf* ), and glucan-binding protein B ( *gbpB* ). 8 9 10 *S. mutans* produces three glucosyltransferases, Gtf-B, -C, and -D. Glucosyltransferases utilize sucrose, which consists of glucose and fructose, as a substrate to synthesize glucan polymers. Glucan plays a crucial role in facilitating *S. mutans* to enhance its adherence to tooth surfaces, binding with other bacteria, and promoting the synthesis of exopolysaccharide (EPS), which is the primary matrix of biofilm formation. Gbp contributes to supporting bacterial attachment to tooth surfaces, depending on sucrose as the foundation of biofilm. 3 11 Inhibiting biofilm formation in *S. mutans* through quorum sensing inhibition can be considered as one of the approaches to prevent caries.

This research will investigate the ability of flora normal bacteria of oral cavity bacteria to inhibit the formation of *S. mutans* biofilm. The bacteria to be used are *Streptococcus gordonii* and *Porphyromonas gingivalis* . According to Wang et al, 12 Challisin produced by *S. gordonii* can inactivate CSP from *S. mutans* . According to Muras et al, 13 *N-* acylated homoserine lactone (AHL) analogs can modify gene expression in polymicrobial biofilm formation. This is demonstrated by the ability of Aii20J (AI-2 inhibitor from Tenacibaculum strain 20J bacteria) to inhibit AI-2 from *S. mutans* in regulating the expression of *gtfB/C/D* in *S. mutans* biofilm formation. *P. gingivalis* is a gram-negative bacterium that communicates through AHL.

## Materials and Methods

### Preparation of *S. mutans, S. gordonii,* and *P. gingivalis*

#### Bacterial Strains and Growth Conditions

This research is a laboratory experimental study conducted at the end of September to the beginning of October 2023. All bacteria used were sourced from the stock at the Research Center of the Faculty of Dentistry, Universitas Airlangga. *S. mutans* (serotype C), *S. gordonii* (ATCC 5165), and *P. gingivalis* (ATCC 33277) bacteria were cultured in trypticase soy broth (TSB) with 5% sucrose. Prepare samples of *S. mutans* (P1), *S. gordonii* (P2), *P. gingivalis* (P3), *S. mutans + S. gordonii* (P4), *S. mutans + P. gingivalis* (P5), and *S. mutans + S. gordonii + P. gingivalis* (P6). They were then incubated under anaerobic conditions at 37°C for 24 hours. After 24 hours, the bacteria solubility will be compared with a McFarland 0.5 solution (10^8 CFU/mL). After that, 150 μL of the bacterial suspension was added to the microplate wells for P1, P2, and P3; 75 μL each for P4 and P5, and 50 μL each for P6. Additionally, 150 μL of TSB was added to the microplate wells as a negative control. Then, the microplate was covered and incubated for 24 hours at 37°C under anaerobic conditions. 14

### Crystal Violet Dye Preparation

After incubating the microplate for 24 hours, the remaining solution in the microplate was discarded. The microplate was washed three times with a phosphate-buffered saline solution at pH 7.4. During each washing, the microplate was tapped to remove the remaining solution from the wells. After the three washes, the microplate was inverted and allowed to air-dry for 10 minutes. 14

### Crystal Violet Assay

Each well was filled with 110 μL 0.4% crystal violet dye solution, and left to stand for 15 minutes. After 15 minutes, the dye solution was pipetted out, and the remaining dye solution was washed away with running water four times. Then, the microplate was allowed to air-dry at room temperature. Once dry, 200 μL of 95% ethanol was added to each well to fix the color bound to the biofilm cells. The microplate was covered and left for 30 minutes. The biofilm was read using a microplate reader (Bio Tek Epoch Microplate Spectrophotometer, Aligent Technologies) at a wavelength of 570 nm. 14

### Methylthiazol Tetrazolium Assay

Solution of methylthiazol tetrazolium (MTT) (Invitrogen by Thermo Fisher Scientific, Life Technologies Corporation Eugene, OR, United States) was added to each well, 15 μL per well. Then, the microplate was covered and incubated for 3 to 4 hours at 37°C under anaerobic conditions. After incubation, 150 μL of dimethyl sulfoxide (Vivantis—ACS Grade, Vivantis Technologies Sdn. Bhd. Malaysia) was added to each well and shaken using a microplate shaker for 5 minutes until the formazan crystals were dissolved. Bacterial metabolism was measured using a microplate reader (Bio Tek Epoch Microplate Spectrophotometer, Aligent Technologies) at a wavelength of 540 nm. 14

### Data Analysis for Crystal Violet Assay

The data from the crystal violet assay was calculated using the following formula to obtain the optical density (OD) values:





The obtained OD values from the above formulas will be categorized into four groups based on their biofilm-producing abilities:

OD isolate ≤ OD C (0) No biofilm forming
OD C < OD isolate ≤ 2 x OD C (+ or 1) weak biofilm forming
2 x OD C < OD isolate ≤ 4 x OD C (++ or 2) moderate biofilm forming
4 x OD C < OD isolate (+++ or 3) high biofilm forming. 14

### Data Analysis for Methylthiazol Tetrazolium Assay

The data from the MTT assay was calculated using the following formula to obtain the OD values:



The obtained OD values from the formula above will be categorized into three groups based on bacterial metabolism:

OD result < 0.75 low cell proliferation
0.75 ≤ OD result ≥ 1.25 normal cell proliferation
OD result > 1.25 increased cell proliferation. 14

The data analysis was conducted to examine if the data is normally distributed and homogeneous as well as to determine if there are significant differences among each treatment. The obtained data were analyzed using the Shapiro–Wilk test to assess the normality of the data distribution, Levene's test for testing the homogeneity of the data, Kruskal–Wallis and Mann–Whitney U test to identify differences between treatments in non-normally distributed data, and one-way analysis of variance and posthoc Games-Howell test to identify differences between treatments in nonhomogeneous data.

## Results

### Biofilm Formation by Crystal Violet

The crystal violet readings in Table 1 indicate the amount of biofilm produced. The control falls into the no biofilm forming category. P3, P4, and P5 fall into the weak biofilm forming category. P1, P2, and P6 fall into the high biofilm forming category.

**Table 1:** Results of crystal violet assay and OD value calculation

Absorbance	Treatment						
	Control	P1	P2	P3	P4	P5	P6
Average	0.155	1.529	1.699	0.662	0.641	0.672	1.461
OD value	−0.089	1.285	1.455	0.418	0.397	0.428	1.217
Interpretation	NBF	HBF	HBF	WBF	WBF	WBF	HBF

Abbreviations: HBF, high biofilm forming; NBF, no biofilm forming; OD, optical density; WBF, weak biofilm forming.

### Bacterial Metabolic by Methylthiazol Tetrazolium

The MTT readings in Table 2 indicate bacterial metabolism abilities. The control falls into the low cell proliferation category. P3 and P5 fall into the normal cell proliferation category. P1, P2, P4, and P6 fall into the increased cell proliferation category.

**Table 2:** Results of MTT assay and OD value calculation

Absorbance	Treatment						
	Control	P1	P2	P3	P4	P5	P6
Average	0.555	1.34	1.524	1.031	1.449	1.195	1.539
OD value	0	1.285	1.469	0.976	1.394	1.14	1.484
Interpretation	LCP	ICP	ICP	NCP	ICP	NCP	ICP

Abbreviations: ICP, increased cell proliferation; LCP, low cell proliferation; MTT, methylthiazol tetrazolium; NCP, normal cell proliferation; OT, optical density.

## Discussion

Biofilm has been proven to be related to and plays a vital role in the occurrence or worsening of infectious diseases. 1 *S. mutans* is a major bacterium responsible for causing dental caries and has the ability to form biofilm on the tooth surface. The formation of biofilm by *S. mutans* is crucial for its survival and also contributes to the development of caries if the biofilm persists and continues to grow on the tooth surface. 5

Biofilm formation of *S. mutans* occurs through quorum sensing mediated by CSP via the two ComDE. The *comC* gene codes for ComC (the precursor of CSP), which is then processed by the ABC transporter complex (ComAB) to produce 21-CSP (a 21-amino acid polypeptide). 21-CSP is cleaved into 18-CSP by SepM (a membrane-localized protease). 18-CSP binds to the histidine kinase receptor (ComD), initiating phosphorylation for the activation of the regulator receptor (ComE). Activated ComE regulates the transcription of specific target genes involved in biofilm formation, including glucosyltransferase B/C/D ( *gtf B/C/D* ), fructosyltransferase ( *ftf* ), and glucan-binding protein B ( *gbpB* ). 8 9 10

*S. mutans* produces three glucosyltransferases, Gtf-B, -C, and -D. Glucosyltransferases utilize sucrose composed of glucose and fructose as substrates to synthesize glucan polymers. GtfB synthesizes insoluble glucan rich in α(1–3) linkages to bind with other bacteria and support bacterial accumulation, GtfC produces soluble glucan rich in α(1–6) linkages and insoluble glucan to enhance bacterial adhesion to tooth surfaces. GtfD produces soluble glucan, also known as dextran, and serves as a primer for Gtf-B synthesis, increasing EPS synthesis. Glucan polymers, especially insoluble glucan rich in α(1–3) linkages, are the main matrix of biofilm plaque. 3 11

Changes in the OD values from the crystal violet and MTT assay each indicate alterations in biofilm formation ability and metabolic activity. Changes in biofilm formation ability and metabolism are associated with alterations in the quorum sensing mechanism responsible for biofilm formation and metabolism. Therefore, changes in OD values reflect alterations in the quorum sensing process.

Based on the research results, the treatment of single species *S. mutans* showed an OD value of 1.285 in both crystal violet and MTT assays. Meanwhile, in the dual species treatment of *S. mutans + S. gordonii* , there was a decrease in polymicrobial biofilm formation (0.397) with increased metabolism (1.394). The reduction in polymicrobial biofilm formation in the *S. mutans + S. gordonii* treatment aligns with the research by Wang and, Kuramitsu and Wang et al, 15 16 which demonstrate that Challisin encoded by the *sgc* gene from *S. gordonii* can inactivate CSP produced by *S. mutans* , leading to the inactivation of ComD. Inactivation of ComD results in ComE inactivation, which in turn affects the inactivation of *gtfB/C/D* , *ftf* , and *gbpB* genes. The *gtfB/C/D* genes are responsible for producing glucosyltransferase enzymes that convert glucose into glucan; the *ftf* gene is responsible for producing fructosyltransferase enzymes that convert fructose into fructan. Glucan and fructan play roles in bacterial adhesion to tooth surfaces, bacterial binding to other bacteria, and as the main matrix of biofilm formation (EPS). The *gbpB* gene supports bacterial adhesion to tooth surfaces and serves as the foundation of biofilm. Inactivation of these three genes reduces biofilm production.

The increase in metabolism in *the S. mutans + S. gordonii* treatment does not correspond to the decrease in biofilm formation. This result does not align with the theory that states biofilm formation is the result of various bacterial metabolic processes, such as amino acid, carbohydrate, and glycolipid metabolism. The results of various metabolisms are used to synthesize various components essential for EPS production during biofilm formation, including amino acids, sugars, lipids, uridine, and organic acids. 17

The treatment results of *S. mutans + P. gingivalis* show a significant decrease in biofilm formation (0.428) with reduced metabolism (1.14). This result is consistent with the research by Muras et al which demonstrates that AHL analogs can modify gene expression in polymicrobial biofilm formation, 13 supported by, 18 which proves the ability of Aii20J (AI-2 inhibitor from Tenacibaculum strain 20J bacteria), an AHL lactonase from Tenacibaculum strain 20J bacteria, to inhibit AI-2 from *S. mutans* in regulating the expression of *gtfB/C/D* in *S. mutans* biofilm formation. Therefore, the decrease in biofilm formation in the treatment of *S. mutans + P. gingivalis* may occur due to the AHL's ability produced by *P. gingivalis* to inhibit *S. mutans* metabolism in biofilm formation.

The treatment results of *S. mutans + S. gordonii + P. gingivalis* show a slight decrease in biofilm formation (1.217) with increased metabolism (1.484). The insignificant decrease in this treatment still categorizes the biofilm formation ability as high biofilm forming. This result may occur because *S. gordonii* facilitates the colonization of *P. gingivalis* even without the presence of *F. nucleatum* as a bridging species. FimA and Mfa1 from *P. gingivalis* bind to glyceraldehyde-3-phosphate dehydrogenase and *Streptococcus* SspA/B adhesin or often referred to as *S. gordonii's* Bacterial Adhesion to Receptor (BAR) antigen. Additionally, the interaction of Mfa1 with SspB activates tyrosine phosphorylation signal production (PTK). Increased PTK signals lead to the formation of exopolysaccharides that result in an increase in *P. gingivalis* colonies. Moreover, it has been demonstrated that community development with *P. gingivalis* does not occur in *Streptococcus species* that lack BAR, such as *S. mutans* and *S. intermedius* . 19 Therefore, there is no increase in biofilm production in the treatment of *S. mutans + P. gingivalis* , but there is an increase in biofilm production in the treatment of *S. mutans + S. gordonii + P. gingivalis* .

The results of the treatment with a single species of *P. gingivalis* (0.418) show lower OD values compared to the other single species, *S. mutans* (1.285) and *S. gordonii* (1.455). The low OD value is due to *P. gingivalis* being cultured in TSB media without heme. In media with insufficient heme, *P. gingivalis* grows slowly because iron in the form of heme is essential nutrition for *P. gingivalis* growth. 20

As a suggestion for further research, this study requires additional experiment that can specifically differentiate the biofilm formation of each bacterium using more advanced technology.

## Conclusion

*S. gordonii* and *P. gingivalis* are each capable of inhibiting the formation of *S. mutans* biofilm in polymicrobial environments. Further research is needed regarding the inducer abilities produced by *S. gordonii* and *P. gingivalis* in inhibiting quorum sensing for *S. mutans* biofilm formation in polymicrobial environments. This research effort aims to prevent dental caries.
